# Should non-disclosures be considered as morally equivalent to lies within the doctor–patient relationship?

**DOI:** 10.1136/medethics-2015-103014

**Published:** 2016-07-22

**Authors:** Caitriona L Cox, Zoe Fritz

**Affiliations:** 1 School of Clinical Medicine, University of Cambridge, Cambridge, UK; 2 Department of Acute Medicine, Addenbrookes Hospital, Cambridge, UK; 3 Department of Health Sciences, University of Warwick, UK

**Keywords:** Applied and Professional Ethics, Autonomy, Clinical Ethics, Patient perspective, Philosophical Ethics

## Abstract

In modern practice, doctors who outright lie to their patients are often condemned, yet those who employ non-lying deceptions tend to be judged less critically. Some areas of non-disclosure have recently been challenged: not telling patients about resuscitation decisions; inadequately informing patients about risks of alternative procedures and withholding information about medical errors. Despite this, there remain many areas of clinical practice where non-disclosures of information are accepted, where lies about such information would not be. Using illustrative hypothetical situations, all based on common clinical practice, we explore the extent to which we should consider other deceptive practices in medicine to be morally equivalent to lying. We suggest that there is no significant moral difference between lying to a patient and intentionally withholding relevant information: non-disclosures could be subjected to Bok's ‘Test of Publicity’ to assess permissibility in the same way that lies are. The moral equivalence of lying and relevant non-disclosure is particularly compelling when the agent's motivations, and the consequences of the actions (from the patient's perspectives), are the same. We conclude that it is arbitrary to claim that there is anything inherently worse about lying to a patient to mislead them than intentionally deceiving them using other methods, such as euphemism or non-disclosure. We should question our intuition that non-lying deceptive practices in clinical practice are more permissible and should thus subject non-disclosures to the same scrutiny we afford to lies.

Lying is done with words and also with silence

-Adrienne Rich

## Introduction

In the past, doctors commonly lied to patients: in 1927, Collins concluded, ‘the longer I practice medicine, the more I am convinced that every physician should cultivate lying as a fine art’.^1^ Today, we would not accept such levels of deception from our physicians.

There are, however, more ways than one to mislead: careful manipulations of language can mould our perceptions of a situation, and false impressions can be cultivated through calculated silence. Several recent scandals and court cases have emphasised the need for clinicians to work with increased levels of openness: the Francis report concluded with the need for a ‘duty of candour’ about clinical mistakes.^2^ The court of appeal judged that it was against an individual's human rights (article 8) not to be told about a resuscitation decision,^3^ and the supreme court held that women have a right to information about ‘any material risk’.^4^

Nevertheless, we continue to accept non-disclosure of information that we would be outraged to find a doctor lying about. The puzzling intuition that not telling someone something is somehow more excusable than lying about it—‘a preference for one mode of deception over another’^5^—is rarely subjected to critical scrutiny in the bioethics domain.

Using illustrative hypothetical situations, all based on common clinical practice, we will explore the extent to which we should consider other deceptive practices in medicine to be morally equivalent to lying.

## Euphemism

Two patients, Anderson and Bailey, have myocardial infarctions. After successful angioplasty, both are left with symptoms of congestive cardiac failure: dyspnoea, peripheral oedema and orthopnoea. Their doctors have different ideas about what they should be told.

1. Doctor A wants to be honest and tells Anderson that although the angioplasty was successful in unblocking the affected coronary artery, he is now suffering from ‘heart failure’. This worries Anderson deeply; he becomes very anxious.
2. Doctor B does not want Bailey to worry, so emphasises the successful angioplasty and euphemistically explains that there is ‘a bit of fluid on the lungs, as the heart is not quite pumping strongly enough’. Bailey is content with this explanation and rates the severity of the illness as much lower.

While doctor A was honest, doctor B made effective use of euphemism, occupying an ill-defined middle ground through the employment of neither complete honesty nor outright lies. The manipulation of language produced very different perceptions of the same ailment in different patients. Was doctor B's turn of phrase deceptive? Neither doctor actually spoke a falsehood, yet clearly Anderson received a more accurate picture of the ‘truth’ than Bailey did.

Consider what we mean by ‘truth’, a concept which Bok distinguishes from ‘truthfulness’.^6^ Correspondence theory considers the ‘truth’ to correspond with the actual state of affairs. Some maintain that as this can never really be known, discussions of ‘truth’ are flawed: ‘telling the truth is impossible, there can be no sharp distinction between what is true and what is false’.^7^ The concept of ‘truthfulness’ is perhaps a more meaningful measure by which we might assess our doctors, as it reflects the *intentions* of the speaker.^6^ The ‘truthfulness’ of a statement is not determined by an objective standard of reality; it is rather determined by whether it coheres to the speaker's own beliefs about what is true and is thus intended to impart accurate information. In the above hypothetical, we must accept that as the recovery from the heart failure is uncertain, the doctor cannot definitely be sure that what he says aligns with reality. We can, however, reasonably expect the doctor's statements to reflect his own beliefs about the outcome: just because the full truth is out of reach, the same cannot be said for truthfulness. If Doctor B intended to mislead with his explanation—even if his motivations were good—he was in a sense not as ‘truthful’ as doctor A.

To keep a secret is ‘to block information about it, or evidence of it from reaching that person, and to do so intentionally’.^8^ Doctor B's euphemism is a form of secrecy, as it prevents Bailey from appreciating the severity of the illness. Bok argues that secrecy is *not* the same as lying, as while lying is prima facie wrong, secrets do not necessarily need to be justified^6^—but that does not mean that all are morally permissible. Secrecy and lying are not identical, but a tenuous border exists between the two concepts, which are ‘woven by the existence of a common antonym: truth’.^9^

Whether Doctor B's euphemism was justified is not at stake; we rather question whether his actions should be viewed as morally equivalent to lying. The information is distorted with an intent—benevolent in nature or otherwise—to mislead; doctor B may reassure himself that he has not technically lied, but such reassurance seems hollow when we consider critically how his choice of words created a false impression.

## Degrees of disclosure

Three patients—Cook, Dobbs and Evans—all suffered pneumonias which resolved with antibiotics. In six weeks' time, they were scheduled for chest X-rays:

1. Cook is told it is to ‘check the pneumonia has cleared up’.
2. Dobbs is told it is to check the pneumonia has cleared and to detect underlying malignancy.
3. Evans is told the same as Dobbs. He is also told that while a CT scan might be more accurate at detecting a malignancy, a chest X-Ray is recommended because of the CT radiation risk.

No one has been lied to, yet Cook has not been as fully informed as Evans. What exactly does one have to disclose, and in how much detail, in order to ensure ‘truthfulness’? In the hypothetical, Cook has been deceived by the non-disclosure, as he does not have a full appreciation of the situation: the doctor has been less ‘truthful’ to him. Yet, while an assertion that to be truthful doctors should disclose all the information seems simple, the practical reality of what this entails is more complex. Theorising that doctors should not withhold information from patients is meaningless unless there is clarity over exactly what information should not be withheld.

The relevance of the information is crucial. If the information concealed is irrelevant to the situation, then its non-disclosure is not problematic: we would not criticise a doctor for withholding from Cook the colour of his socks. Benn has stated that concealment only becomes deceptive if people would reasonably expect the agent to reveal the information to those who do not know it.^10^ Similarly, Bok emphasises the importance of whether information is owed to a subject in assessing deception in the context of placebos.^6^ It is perhaps thus the *relevance* of the information to the patient which is of real moral importance, not whether the information is withheld through silence or distorted through falsehood.

If we accept that intentionally withholding relevant information is deceptive, we must consider how one might practically define ‘relevance’. Legally, attempts have been made to define the limits of disclosure. The earlier ‘Professional Standard’ has been challenged by a ‘Patient-Orientated Standard’, according to which doctors must tell patients what a reasonable person in that situation would want to know.^11^ A subjective patient-orientated standard, in which information that a *particular* patient would find useful must be disclosed, has been incorporated into a ‘decision checklist’ to help clinicians determine whether it might be morally acceptable to deceive patients.^12^

Problems with this standard of disclosure have been raised. O'Neill^13^ has noted the impossibility of providing a patient with ‘all’ the relevant information: a doctor could never share with patients every detail influencing their decision-making process, both in terms of information volume and technical complexity. There is more to ethical disclosure than providing patients with the objective medical information: the facts must be presented in ‘such a way that the recipients are able, should they so wish, to understand the consequence of communicated facts’.^14^ The concept of materiality may justify the rejection of sharing overly technical information—if the information is so complex that a patient would not understand it, it will not influence their decision and so disclosure may not be obligated. If we return to the example above, if the patient does not understand what ‘heart failure’ means, full disclosure may be truthful, but not ethical.

Another question remains over the disclosure of information which patients may find distressing: should information be withheld in the name of beneficence or non-maleficence? That the full truth might be harmful to patients is reflected in the legal concept of ‘therapeutic privilege’, in which doctors are excused from disclosing information if it ‘poses such a threat of detriment to the patient so as to become unfeasible or contraindicated from a medical point of view’.^11^ Evidence for bad news actually rendering patients incapable of rational decision-making, or otherwise producing serious harm, is limited. Even if one accepts non-maleficence as important, it is questionable whether withholding distressing information furthers this: one may suffer greater harm as a result of being given false confidence than would be inflicted by a compassionate and honest discussion of the truth.^15^ Moreover, the principle has been challenged recently in UK courts: a judgement held that doctors had breached a patient's human rights when they did not discuss a decision not to attempt resuscitation in the event of her heart stopping.^3^ By not disclosing their decision—a clinical decision which was not disputed—they deprived her of the chance to ask them questions or seek a second opinion. This judgement thus represents a strong curtailing of therapeutic privilege.

These discussions have implications for the nature of the doctor–patient relationship. Some worry that giving patients too much technical information may not just be unhelpful, but unethical: the ‘doctor who merely spreads an array of vendibles in front of the patient and then says “go ahead and choose, it's your life” is guilty of shirking his duty, if not malpractice’.^16^ If doctors become preoccupied with a neutral truth—with giving patients ‘all’ of the medical detail—truth-telling may become a one-way act, with doctors delegating, not sharing, the responsibility for medical decisions.

In sum, consensus on the scope of disclosure remains elusive. Practical difficulties regarding relevance definitions, as well as considerations of implications for the doctor–patient relationship and the logistical implications of talking through multiple variables with every patient, remain problematic. Withholding relevant information can, however, represent an unacceptable deception.

## Lying versus withholding information

Two medical students want to practice inserting cannulas and are directed to two patients, Fisher and Griffiths. Student F tells Fisher she is a student doctor; Fisher enquires whether she has ever done the procedure before. Student F fears that he may not consent to the procedure if he knew the truth, so replies that she has. (She justifies this lie to herself because she has practised on dummies many times and passed the exam with flying colours.) Reassured, Fisher consents. Student G tells Griffiths that she has been told he needs a cannula, but does not explicitly mention that she is a student doctor as she too fears that this may discourage him from consenting. Griffiths wrongly assumes that she has performed the procedure before and happily consents. Both patients consented to the procedure thinking that the students were more experienced than they were—so has student F acted more wrongly by lying than student G has by merely ‘not telling’?

Reaching a firm conclusion about the morality of lying using traditional deontological, utilitarian or virtue theories alone is unsatisfying at best. Bok provides a practical perspective, encouraging consideration of lies from the perspective of both the deceiver and the deceived, emphasising the need to preserve trust within society. She adopts a ‘principle of veracity’, noting the moral imbalance in that ‘lying requires a reason, whilst truth-telling does not’.^6^ Bok concedes that lies may be sometimes justified, proposing a ‘Test of Publicity’.^6^ One must consider

1. possible truthful alternatives,
2. the moral arguments for and against the lie,
3. what a public jury of reasonable persons would say.

Using this model, we might conclude that we have a prima facie reason not to lie which is not, however, absolute.

Lying within the doctor–patient relationship has received further analysis. Paternalism permitted a degree of benevolent deceit; yet commonly cited justifications are unconvincing. The perception that patients would rather not hear bad news has been repeatedly demonstrated to be false: as a literature review concluded, ‘failure to disclose… on the grounds that significant numbers of patients prefer not to know is untenable’.^17^ Higgs notes the concept to be laughably paternalistic, asking us to consider an accountant who does not tell his client of their bankruptcy as they would ‘rather not know’.^18^ While a minority of patients may genuinely rather not be told, to assume the same for all patients would be deeply flawed. Beneficence as a justification for lying assumes that doctors are the most competent ‘assessors of happiness’.^19^ Patients make choices based not merely upon technical knowledge, but on a myriad of factors, about which there is no reason to assume physician judgement superiority.^19^

Even if we accept that doctors have a duty of beneficence which can be achieved through lying, we need not assume that this principle is superseding. Many have argued that this would demonstrate an unacceptable lack of respect for autonomy. When lying, the doctor is ‘making a unilateral decision to deny the patient the opportunity to exercise his or her autonomy’.^14^ To engage in rational deliberation, patients must have accurate information—thus decisions based on false information are not truly autonomous. A discussion of autonomy in bioethics is beyond the scope of this essay, but it has been emphasised by many modern ethicists, notably O'Neill.^13^

In sum, the justifications traditionally employed to excuse lying to patients are largely unconvincing; while lying is not absolutely wrong in all situations, doctors are rarely permitted to lie to their patients. In the above hypothetical, few would argue that student F's lie was morally permissible: it was clearly told for reasons of self-preservation, and it denied the patient the opportunity to autonomously provide consent. A more contentious issue is whether student G's actions were also wrong, and if so, whether they were equally so. The acceptance of a morally significant distinction between lying about something and not disclosing it requires closer examination.

Some argue that non-disclosure is not as deceptive as lying: the agent does not create new false beliefs, but merely fails to correct existing ones.^20^ Certainly, in some circumstances, the liar—by actively providing misleading information—steers someone further from the truth than the non-discloser. Yet, in many clinical contexts, this does not fully apply. In the above hypothetical, both Fischer and Griffiths ultimately ended up with the same false belief—and were thus equally far from the truth—regardless of whether they were actively provided with misleading information. Saul rejects that non-disclosure is more permissible as the responsibility for the belief is shared between the deceiver and the deceived, as this does not alter wrongness of the deception.^5^ Consider the mugging victim who goes out alone at night: although the victim arguably carries some of the responsibility, this does not in itself diminish the intrinsic badness of the mugging act.^5^ Jackson states it is only within a framework of utilitarianism that we should consider lying and non-lying forms of deception as equivalent;^21^ Benn argues that it is possible to reject utilitarianism yet still support a significant difference on the basis of other principles (such as the importance of intentions).^10^ Both argue that lying is more harmful than non-lying deception to community trust;^10^ ^21^ yet withholding information can arguably be as damaging as lying, as it undermines the principle of honest communication at the core of the fiduciary doctor–patient relationship.^15^ Benn's argument that our psychological aversion to lying supports the view that it is worse than concealment is unconvincing.^10^ It is thus difficult to justify a claim that lying is itself any worse than not telling, based on both acts' intrinsic properties.

Medical students and trainees—particularly surgical—are rarely encouraged to be totally honest about lack of experience. Yet studies have shown that patients want to know if it is the clinician's first time performing a procedure and about their physician's level of training more generally.^22^ There thus exists a tension between what patients want to be informed of and what tends to be disclosed in practice. The American law remains inconsistent: some cases have upheld the need to disclose the level of surgeon experience, while others have disagreed.^23^

If the patient wishes to be appraised, why should withholding the information be any more permissible than lying about it? One way to approach this problem might be to employ Bok's ‘Test of Publicity’, applying a similar moral criteria to non-disclosure as has been used to analyse lying. In this hypothetical, student G's non-disclosure is impermissible on all components of Bok's test: truthful alternatives are clearly available, the moral reasons for the non-disclosure are questionable at best and a jury of reasonable persons would most likely demand the information's disclosure. Here, it seems clear that non-disclosure is as unacceptably deceptive as lying.

## Intentions and consequences

Two young patients are in hospital in late December. Hughes asks doctor H whether he will be home for Christmas—not wanting to upset the boy, doctor H replies ‘Yes’, even though he is sure he will not. Josephs does not ask doctor J such a question, but instead talks excitedly about being home for Christmas dinner. Doctor J also does not want to upset Josephs, so does not mention that he thinks this unrealistic. Is it reasonable to attempt to draw any sort of moral distinction between the two doctors' actions?

Consider how this problem relates to the debate surrounding the acts/omissions distinction, according to which, ‘failure to perform an act, with certain foreseen bad consequences of that failure, is morally less bad than to perform a different act which has the identical foreseen bad consequences’.^24^ That a morally significant distinction can be made has been criticised compellingly by Rachels, through consideration of the agent's intentions in his Smith/Jones thought experiment.^25^ Similarly, when one considers doctors H and J's intentions in this hypothetical, a moral distinction seems less convincing.

Imagine that doctor H is evil and enjoys crushing children's dreams. Doctor H thus tells Hughes that he will most likely not be home in time for Christmas, causing him distress. In contrast, doctor J cares deeply about his patient, and thus tries to protect him by avoiding the topic. Doctor H was more honest, yet doctor J's actions seem more justified, suggesting that the motivations of the doctors carry the real moral weight. To deceive is to intentionally mislead; if one deceives another, either through speaking a falsehood or by withholding relevant information, then the morality of the action is equivalent when the motivation is also the same. This is a perspective that Higgs has advanced, arguing that the morality of doctors' actions is determined by whether they are intended to mislead, irrespective of the means employed.^18^

Now imagine that both patients actually recover sufficiently to get home by Christmas. Does this alter the rightness or wrongness of the doctors' respective actions? If the patients had not gone home and had been very disappointed, we intuitively feel that the doctors' actions were more wrong. This supports the utilitarian view that that the consequences help to define the morality of the doctors' communications.

These hypotheticals suggest that both the intentions and consequences of the doctors' actions are of more ethical relevance than the distinction between one doctor lying and the other ‘not telling’. In the initial scenario, it seems arbitrary to claim that doctor H acted any more immorally than doctor J. The intentions for, and consequences of, both actions were identical, thus should they not be judged as such?

## Conclusion

We tend to accept that outright lying is in general wrong: doctors are only very rarely permitted to lie to their patients. What is less clear is whether withholding information is wrong, and if so, whether it is equally wrong. Through a discussion of hypothetical cases, we conclude that it is flawed to draw a morally significant distinction between intentionally misleading someone by providing them with false information on a topic and omitting to tell them something of relevance about this topic. This is particularly true if the intentions and consequences of both actions are the same.

We have not argued that non-disclosures are always wrong: as is the case with lying, in some circumstances they may be justified. What we conclude is that we should treat decisions to intentionally withhold relevant information as equally morally problematic as decisions to lie. It follows that we should adopt a more stringent policy regarding non-disclosures in clinical practice, subjecting them to the same analysis we might give to decisions to lie. For example, it may be useful to apply measures such as Bok's ‘Test of Publicity’ not just to instances of doctor's lying, but also to cases in which doctors deceive patients through euphemism or non-disclosure. Using such a model to scrutinise modern clinical practice, it becomes clear that there are a number of areas in which disclosure is not routine, but perhaps should be. Such areas are thus in need of more detailed exploration.
